# The Mental Health Impact of COVID-19 Racial and Ethnic Discrimination Against Asian American and Pacific Islanders

**DOI:** 10.3389/fpsyt.2021.708426

**Published:** 2021-11-17

**Authors:** Sasha Zhou, Rachel Banawa, Hans Oh

**Affiliations:** ^1^Department of Public Health, Wayne State University, Detroit, MI, United States; ^2^Fielding School of Public Health, University of California, Los Angeles, Los Angeles, CA, United States; ^3^Suzanne Dworak Peck School of Social Work, University of Southern California, Los Angeles, CA, United States

**Keywords:** racism, COVID-19, pandemic (COVID-19), Asian American (AA), discrimination, mental health, college

## Abstract

Hate crimes against Asian American/Pacific Islanders (AAPIs) have surged in the United States during the COVID-19 pandemic to alarming new levels. We analyzed data from the Healthy Minds Study, and found that COVID-19 related racial/ethnic discrimination was associated with greater odds of having depression, anxiety, non-suicidal self-injury, binge drinking, and suicidal ideation among AAPI university students (*N* = 1,697). Findings suggest that the COVID-19 pandemic precipitated discrimination, which has been linked to mental health problems, calling for more preventive interventions to address the AAPI population, especially given their low rates of formal treatment utilization.

## Introduction

Racism against Asian American Pacific Islanders (AAPI) is not a new phenomenon in the United States, but reports of discrimination and hate crimes against this community have surged to new heights during the COVID-19 pandemic. The term “AAPI” refers to any individuals living in the United States who identify as Asian or Pacific Islander, including both US citizens and non-US citizens. According to the Pew Research Center (1), about 40% of Asian American adults reported that other people were visibly uncomfortable around them since the start of the pandemic. According to the Center for the Study of Hate and Extremism (2), anti-Asian hate crimes increased by almost 150% across 16 of the country's largest cities in the year 2020. And between March 19, 2020 and February 28, 2021, the Stop AAPI Hate reporting center documented 3,795 hate incidents, ranging from online harassment to physical assault (3). One example of these hate incidents occurred at a metro station in Washington, DC when a man punched and/or pushed two Asian individuals while yelling racial slurs; this same man was later seen entering a Chinese tea store and pepper spraying the owner (3). The list of hate crimes is harrowing and continues to grow (4); we should note that many hate crimes go unreported. Much of this racism has been fueled by a xenophobic narrative that AAPI's are somehow responsible for the COVID-19 pandemic, underscoring a long-held view that AAPIs are perpetual foreigners who do not belong in the country (5). This racialization of COVID-19 has the potential to produce long-lasting effects on attitudes toward AAPIs, which is alarming since a substantial body of research has linked racial discrimination to adverse mental health outcomes as well as lower use of formal psychiatric treatment (6). In this study, we analyzed a sample of AAPI university students from across the country to examine the associations between COVID-19 related racial/ethnic discrimination and mental health outcomes during the pandemic.

## Methods

We analyzed data the 2020 Healthy Minds Study (HMS), which is a cross-sectional, web-based survey examining mental health and related factors in students enrolled at one of 29 universities. Among participating institutions, eight are Associate's Colleges, three are Baccalaureate Colleges, four are Master's Colleges and Universities, and 14 are Doctorate-granting Universities. Six of the colleges and universities are private institutions, and the remaining 23 are public institutions. The HMS is designed to protect the privacy and confidentiality of participants, and has been approved by the Health Sciences and Behavioral Sciences Institutional Review Board at University of Michigan. To further protect respondent privacy, the study is covered by a Certificate of Confidentiality from the National Institutes of Health. The study survey was administered between September through December of 2020.

While COVID-19 related ethnic/racial discrimination was reported by multiple racial/ethnic groups, AAPIs were by far the most impacted. Using the entire sample of respondents who completed the COVID-19 module, we found that being AAPI students were more than 17 times as likely as white students to have experienced racial/ethnic discrimination in the context of COVID-19, adjusting for age and gender (aOR: 17.45; 95% CI: 12.25–24.86). Thus, for the purposes of this report, we restricted our analysis to AAPI students (*N* = 1,697). The mean age of this AAPI sample was 23.78 years old (95%CI: 23.15–24.41), and the majority was cis-gendered women (67.88%; *n* = 1,152).

To adjust for potential differences between responders and non-responders, sample probability weights were applied. HMS obtains administrative data from participating institutions, including gender, race/ethnicity, academic level, and grade point average to construct response weights, equal to 1 divided by the estimated probability of response, using a logistic regression to predict the likelihood of response associated with each variable.

Respondents were asked a single binary item (yes/no): *As a result of the COVID-19 pandemic, have you experienced any discriminatory or hostile behavior due to your race/ethnicity (or what someone thought was your race/ethnicity)*? We examined this question in relation to several mental health outcomes: depression, anxiety, binge drinking, non-suicidal self-injury, and suicidal ideation. We conducted multivariate logistic regression models, adjusting for age and gender, to assess the impact of discrimination on these mental health outcomes.

We focused on binary measures of mental health because most of the measures have been validated based on standard cutoffs. We examined symptoms of depression using the Patient Health Questionnaire (PHQ-9). The PHQ-9 has been validated as internally consistent and highly correlated with clinical diagnosis, including among people of color (7). We used the standard cut off of >15, indicating moderately severe to severe depression. Anxiety was measured using the Generalized Anxiety Disorder-7 scale which has been used in racially diverse samples (8). We used the standard cut off of a score higher than 10, which has been shown to have high specificity and sensitivity in indicating moderate to severe anxiety (8). Binge drinking was assessed dichotomously, with a positive endorsement if respondents reported binge drinking (4 if female and 5 if male alcoholic drinks in a row) at least once during the past 2 weeks. This item originated from the College Alcohol Study and validated in college populations (9). Non-suicidal Self-injury was dichotomized using a positive endorsement of the following item developed for the Healthy Minds Study: In the past year, have you ever done any of the following intentionally: Cut myself, burned myself, punched or banged myself, scratched myself, pulled my hair, bit myself, interfered with wound healing, carved words or symbols into skin, punched or banged an object to hurt myself, other? Suicidal ideation was assessed using the single binary item (yes/no): In the past year, did you ever seriously think about attempting suicide?

## Results

**Figure 1:**
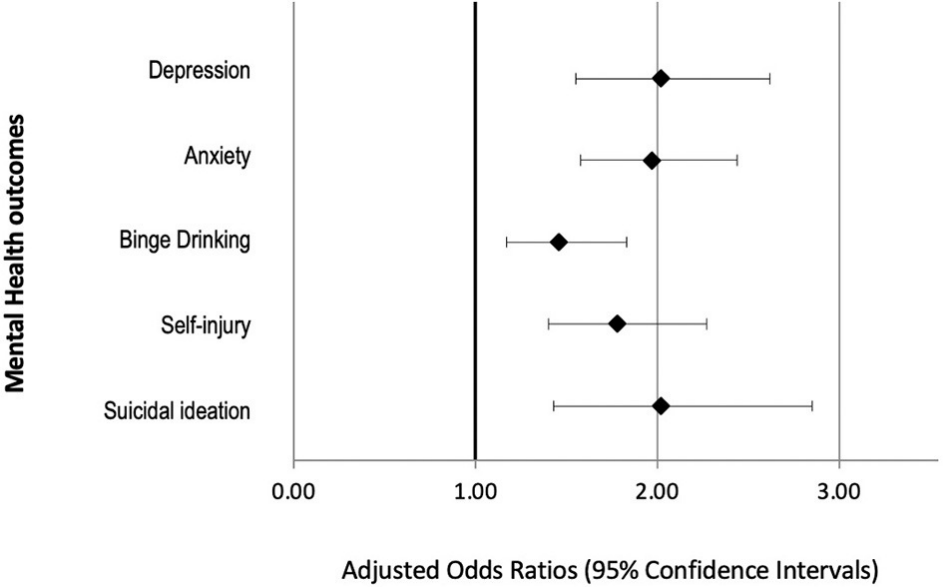
Adjusted odds ratios depicting associations between COVID-19 related racial discrimination and mental health outcomes among Asian American/Pacific Islander students in the Health Minds Study, September–December 2020. All models adjusted for age and gender.

Among the AAPI students, over a quarter reported experiencing COVID-19 related racial/ethnic discrimination (Table 1). Over two-thirds of respondents who endorsed this item met the criteria for at least one clinically significant mental health condition. Using multivariable logistic regression models, we found that COVID-19 related racial/ethnic discrimination was associated with greater odds of having moderately severe or severe depression, moderate to severe anxiety, any binge drinking over the past 2 weeks, non-suicidal self-injury, and suicidal ideation, adjusting for age and gender (Table 2). Findings are summarized in Figure 1.

**Table 1:** Associations between COVID-19 related racial/ethnic discrimination and mental health outcomes among American/Pacific Islander students from the Healthy Minds Study, September–December 2020.

	**Descriptive statistics**			
	**Total** **textitN = 1,697**	**No discrimination** ***N*** **= 1,262 (74.37%)**	**Discrimination** ***N*** **= 435 (25.63%)**	***P*** **-value**
Depression				
No	1,395 (83.88%)	1,073 (86.95%)	322 (75.06%)	<0.001
Yes	268 (16.12%)	161 (13.05%)	107 (24.94%)	
Anxiety				
No	948 (76.58%)	1,203 (72.30%)	255 (59.86%)	<0.001
Yes	290 (23.42%)	461 (27.70%)	171 (40.14%)	
Binge drinking				
No	1,372 (80.85%)	1,039 (82.33%)	333 (76.55%)	<0.001
Yes	325 (19.15%)	223 (17.67%)	102 (23.45%)	
Non-suicidal self-injury				
No	1,364 (81.68%)	1,050 (84.54%)	314 (73.36%)	<0.001
Yes	306 (18.32%)	192 (15.46%)	114 (26.64%)	
Suicidal ideation				
No	1,545 (91.26%)	1,172 (93.09%)	373 (85.94%)	<0.001
Yes	148 (8.74%)	87 (6.91%)	61 (14.06%)	

*P-values reflect t-test for continuous variables and Chi^2^ test for binary/categorical variables*.

*(1) Depression was measured using the Patient Health Questionnaire 9, which is a scale ranging from 0 to 27. We dichotomized the scale to reflect individuals who reported a score higher than 15, indicating moderately severe to severe depression*.

*(2) Anxiety was measured using the Generalized Anxiety Disorder 7, which is a scale ranging from 0 to 21. We dichotomized this scale to reflect individuals who reported a score of higher than 10, indicating moderate to severe anxiety*.

*(3) Binge drinking was assessed using the item: Over the past 2 weeks, did you have 4 (if female)/5 (if male) or more alcoholic drinks in a row?*.

*(4) Self-injury was assessed using the item: In the past year, have you ever done any of the following intentionally: Cut myself, burned myself, punched or banged myself, scratched myself, pulled my hair, bit myself, interfered with wound healing, carved words or symbols into skin, punched or banged an object to hurt myself, other?*.

*(5) Suicidal ideation was assessed using the single binary item (yes/no): In the past year, did you ever seriously think about attempting suicide?*

**Table 2:** Multivariable logistic regression models showing the relations between COVID-19 related racial/ethnic discrimination and mental health outcomes among American/Pacific Islander students from the Healthy Minds Study, September– December 2020.

	**Multivariable logistic regression models**
	**aOR [95% CI]**
Depression	
No	1.00
Yes	2.02 [1.55–2.62]
Anxiety	
No	1.00
Yes	1.97 [1.58–2.44]
Binge drinking	
No	1.00
Yes	1.46 [1.17–1.83]
Non-suicidal self-injury	
No	1.00
Yes	1.78 [1.40–2.27]
Suicidal ideation	
No	1.00
Yes	2.02 [1.43–2.85]

## Discussion

While considerable literature has shown that exposure to racial/ethnic discrimination increases odds of having mental health problems (6, 10–12), a growing body of literature has documented the increase in discrimination and stigmatization during the pandemic, especially against Asians across the globe (13, 14). The current study builds on emerging literature by showing the potential mental health effects of racial/ethnic discrimination specifically in the context of COVID-19 pandemic among Asian American college students, which should be factored into the overall health and economic burden of the pandemic. To the best of our knowledge, the specific impact of pandemic-related discrimination has not been studied at the national level, in young and emerging adults who identify as Asian American.

These findings should be interpreted bearing in mind that racial/ethnic discrimination was self-reported, which is prone to both under- and over-reporting (15). Moreover, the study used a non-probability sampling strategy that yielded a response rate of 14%, which is admittedly low but common for these types of online surveys (8). We did however use sample probability weights to adjust for non-response using the following administrative data on full student populations: gender, race/ethnicity, academic level, and Grade Point Average. Still, it remains to be seen whether these associations are generalizable to the larger AAPI population and global Asian population; it is possible that the associations may be even stronger outside of the university context, especially among immigrants with limited English proficiency.

Historically, based on data collected in prior years of the HMS survey, less than a third of AAPI students with a clinically significant mental health condition are engaged in mental health treatment, which is the lower than other racial groups (16). Preventive interventions are needed to eliminate this treatment gap. Undoubtedly, anti-Asian discrimination and hate crimes continue to devastate individuals and communities across the world, and so as AAPI researchers, we urge our colleagues and institutions to speak out publicly against this hatred, to design interventions that mitigate the pernicious effects of racism on population health, and to call for the removal of barriers that prevent racial and ethnic minorities from accessing appropriate mental health treatment.

## Data Availability Statement

Publicly available datasets were analyzed in this study. This data can be found here: https://healthymindsnetwork.org/hms/.

## Ethics Statement

The studies involving human participants were reviewed and approved by University of Michigan. The patients/participants provided their written informed consent to participate in this study.

## Author Contributions

SZ contributed to the data collection and writing. RB contributed to the writing and editing. HO contributed to the writing and conceptualization. All authors contributed to the article and approved the submitted version.

## Conflict of Interest

SZ is an investigator for the Healthy Minds Study. The remaining authors declare that the research was conducted in the absence of any commercial or financial relationships that could be construed as a potential conflict of interest.

## Publisher's Note

All claims expressed in this article are solely those of the authors and do not necessarily represent those of their affiliated organizations, or those of the publisher, the editors and the reviewers. Any product that may be evaluated in this article, or claim that may be made by its manufacturer, is not guaranteed or endorsed by the publisher.

## References

[1] Pew Research Center. Many Black and Asian Americans Say They Have Experienced Discrimination Amid the COVID-19 Outbreak. (2020). Available online at: https://www.pewresearch.org/social-trends/wp-content/uploads/sites/3/2020/07/PSDT_07.01.20_racism.covid_Full.Report.pdf (accessed March 19, 2021).

[2] Center for the Study of Hate and Extremism California State University San Bernardino. Fact Sheet: Anti-Asian Prejudice. (2020). Available online at: https://www.csusb.edu/sites/default/files/FACT%20SHEET-%20Anti-Asian%20Hate%202020%203.2.21.pdf (accessed March 19, 2021).

[3] Jeung R Horse AY Popovic T Lim R. 2020-2021 National Report. Stop AAPI Hate. (2021). Available online at: https://secureservercdn.net/104.238.69.231/a1w.90d.myftpupload.com/wp-content/uploads/2021/03/210312-Stop-AAPI-Hate-National-Report-.pdf (accessed March 19, 2021).

[4] Cai W Burch ADS Patel JK. Swelling anti-Asian violence: who is being attacked where. New York Times. (2021). Available online at: https://www.nytimes.com/interactive/2021/04/03/us/anti-asian-attacks.html (accessed April 9, 2021).

[5] Li Y Nicholson HL Jr. When “model minorities” become “yellow peril” —Othering and the racialization of Asian Americans in the COVID-19 pandemic *Sociol Compass*. (2021) 15:e12849. 10.1111/soc4.12849 33786062 PMC7995194

[6] Lee DL Ahn S. Racial discrimination and Asian mental health: a meta-analysis. Counsel Psychol. (2011) 39:463–89. 10.1177/001100001038179130907605

[7] Huang FY Chung H Kroenke K Delucchi KL Spitzer RL. Using the patient health questionnaire-9 to measure depression among racially and ethnically diverse primary care patients. J Gen Intern Med. (2006) 21:547–52. 10.1111/j.1525-1497.2006.00409.x 16808734 PMC1924626

[8] Spitzer RL Kroenke K Williams JB Löwe B. A brief measure for assessing generalized anxiety disorder: the GAD-7. Arch Intern Med. (2006) 166:1092–7. 10.1001/archinte.166.10.1092 16717171

[9] Wechsler H Davenport A Dowdall G Moeykens B Castillo S. Health and behavioral consequences of binge drinking in college: a national survey of students at 140 campuses. JAMA. (1994) 272:1672–7. 10.1001/jama.1994.03520210056032 7966895

[10] Carter RT Lau MY Johnson V Kirkinis K. Racial discrimination and health outcomes among racial/ethnic minorities: a meta-analytic review. J Multicult Counsel Dev. (2017) 45:232–59. 10.1002/jmcd.1207625855820

[11] Pascoe EA Smart Richman L. Perceived discrimination and health: a meta-analytic review. Psychol Bull. (2009) 135:531. 10.1037/a0016059 19586161 PMC2747726

[12] Schmitt MT Branscombe NR Postmes T Garcia A. The consequences of perceived discrimination for psychological well-being: a meta-analytic review. Psychol Bull. (2014) 140:921. 10.1037/a0035754 24547896

[13] Bhanot D Singh T Verma SK Sharad S. Stigma and discrimination during COVID-19 pandemic. Front Public Health. (2020) 8:829. 10.3389/fpubh.2020.577018 33585379 PMC7874150

[14] Wu C Qian Y Wilkes R. Anti-Asian discrimination and the Asian-white mental health gap during COVID-19. Ethnic Racial Stud. (2020) 44:1–17. 10.2139/ssrn.3626460

[15] Lewis TT Cogburn CD Williams DR. Self-reported experiences of discrimination and health: scientific advances, ongoing controversies, and emerging issues. Annu Rev Clin Psychol. (2015) 11:407–40. 10.1146/annurev-clinpsy-032814-112728 25581238 PMC5555118

[16] Lipson SK Kern A Eisenberg D Breland-Noble AM. Mental health disparities among college students of color. J Adolesc Health. (2018) 63:348–56. 10.1016/j.jadohealth.2018.04.014 30237000

